# Evaluating genome architecture of a complex region via generalized bipartite matching

**DOI:** 10.1186/1471-2105-14-S5-S13

**Published:** 2013-04-10

**Authors:** Christine Lo, Sangwoo Kim, Shay Zakov, Vineet Bafna

**Affiliations:** 1 Department of Computer Science and Engineering, University of California, San Diego, CA, USA

## Abstract

With the remarkable development in inexpensive sequencing technologies and supporting computational tools, we have the promise of medicine being personalized by knowledge of the individual genome. Current technologies provide high throughput, but short reads. Reconstruction of the donor genome is based either on *de novo *assembly of the (short) reads, or on mapping donor reads to a standard reference. While such techniques demonstrate high success rates for inferring 'simple' genomic segments, they are confounded by segments with complex duplication patterns, including regions of direct medical relevance, like the HLA and the KIR regions.

In this work, we address this problem with a method for assessing the quality of a predicted genome sequence for complex regions of the genome. This method combines two natural types of evidence: sequence similarity of the mapped reads to the predicted donor genome, and distribution of reads across the predicted genome. We define a new scoring function for read-to-genome matchings, which penalizes for sequence dissimilarities and deviations from expected read location distribution, and present an efficient algorithm for finding matchings that minimize the penalty. The algorithm is based on a formal problem, first defined in this paper, called **C***overage ***S***ensitive many-to-many min-cost bipartite ***M***atching *(CSM). This new problem variant generalizes the standard (one-to-one) weighted bipartite matching problem, and can be solved using network flows. The resulting Java-based tool, called SAGE (**S***coring function for ***A***ssembled ***GE***nomes*), is freely available upon request. We demonstrate over simulated data that SAGE can be used to infer correct haplotypes of the highly repetitive KIR region on the Human chromosome 19.

## Introduction

The inexorable drop in costs and rise in throughput of DNA sequencing is driving a future in which every individual person will have their genome sequenced, perhaps multiple times in their lifetimes [1]. Current high throughput technologies produce sequenced read fragments from donor genomes, which are then used for inferring the complete genomic sequence. The main algorithmic approaches for inferring a donor genome from a set of its sequenced reads are either based on *de novo assembly *[2,3], i.e. producing a parsimonious super-string that approximately contains most reads as its substrings, or based on *mapping *approaches [4-6], in which the algorithm takes the read set and a previously sequenced reference genome (or a set of reference genomes), maps the reads to the reference, and uses the identified similarities and variations in order to predict the donor genome.

**Figure 1:**
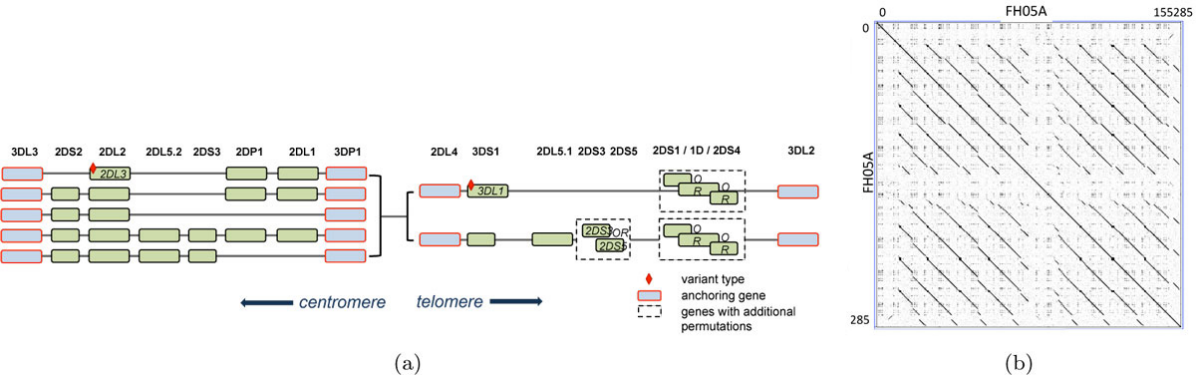
**KIR region**. (a) Variability of gene architecture in KIR haplotypes (derived from Hsu [10]). (b) Complex repeat structure in a KIR haplotype, as observed by a dot-plot of FH05A against FH05A. The different genes all show significant sequence similarity. Dot-plot prepared using Gepard [22].

While the accuracies of sequencing technologies keep improving and their usage costs keep decreasing, many of them still produce reads of relatively short lengths. Reconstruction of repetitive genomic regions using the mentioned approaches is considered more challenging, due to the fact that short reads may be de-novo assembled, or mapped to the reference, in multiple ambiguous manners. The difficulty even increases for diploid genomes, limiting the investigation of many important genomic regions, such as the killer cell immunoglobulin like receptor (KIR) region (located in humans within the 1Mb Leucocyte Receptor Complex 19q13.4, see Figure 1b), the 3.6Mbp Human Leucocyte Antigen (HLA) region and others, which exhibit highly repetitive sequences and extensive polymorphisms.

Here, we address the problem of *assessing the quality *of a donor genome prediction given the set of its sequenced reads, confronting difficulties related to genomic regions of repetitive nature. We present a prediction quality measure a prediction quality measure which is independent of the approach used for generating the prediction. It combines scoring penalties related to both (a) imperfect alignments of the reads to the predicted region, and (b) deviations between the expected and actual read coverage of segments of the region. Our tool differs from previous ones which compare predictions to a known reference. For example, tools that evaluate the quality of de-novo assemblies [7] rely on comparing assembled genomes to known references. Mapping tools [8,9] can be used to provide a naive scoring function comparable to SAGE by summing up the best alignment score of each read. This naive scoring function only optimizes the alignment of the reads and does not take into account read coverage. Our results show the advantage of simultaneously optimizing the combined alignment and coverage score by comparing our tool to the naive approach.

In order to evaluate the new cost function, we applied it to the KIR, a hyper-variable region known to be important for the immediate immune response in humans and higher mammals [10]. The KIR region is challenging to reconstruct from sequence read fragments due to its variable gene architecture (Figure 1a) and repetitive nature (Figure 1b). We show that our scoring function allows us to correctly identify KIR haplotype templates in diploid genomes, differentiating correct predictions form incorrect ones based on their computed score, while the naive approach fails in many cases to predict the correct template.

Our cost function for evaluating donor genome predictions is based on a new variant of a bipartite matching problem, entitled *Coverage Sensitive many-to-many min-cost bipartite Matching *(CSM), which is a many-to-many generalization of the classical min-cost (or max-weight) bipartite matching problem [11,12]. The formal definition of the CSM problem is given in the next section. While in general CSM is NP-Hard (see Additional File 1), we show a special "convexed" case for which CSM can be efficiently solved by reducing it to a network flow problem, similar to many other variants of bipartite matching problems [12]. Optimal matching/flow algorithms were recently used by several related works to predict structural variations between genomes. Examples to such works include [13], in which min-cost flow was used to call copy number variations between a reference and a donor genome, [14], which used maximum-weight matching in order to reconstruct breakpoint sequences in long genomic insertions, and [15], which used maximum-flow in order to apply a post-process refinement of simultaneous detection of structural variations in multiple genomes.

## Coverage Sensitive many-to-many min-cost bipartite Matching (CSM)

The CSM problem is a many-to-many generalization of the classical min-cost bipartite matching problem [12]. We describe the problem in an abstract setting, and cast it to a read alignment problem in the next section.

**Figure 2:**
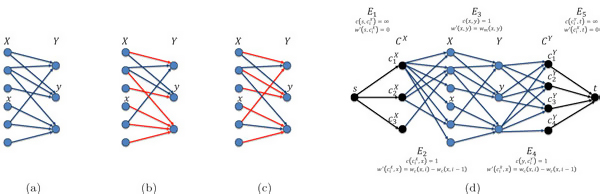
**Matching instance and its reduction to a cost flow network**. (a) A bipartite graph corresponding to sets *X *and *Y*. In our particular application, *X *represents a set of reads and *Y *represents a set of genomic segments, where the expected coverage of each read is one and segments are expected to be uniformly covered. Each read *x *∈ *X *potentially maps to multiple segments, illustrated by the edges in the graph. An edge (*x*, *y*) has the weight *w_m _*(*x*, *y*), reflecting the best similarity between read *x *and a substring of of the genome starting at segment *y*. (b) and (c) depict two possible *matchings*. In (b), one of the *y *segments is covered by four reads, while the other two segments are covered by one read each. In (c), each segment is covered by two reads. It is possible that the matching in (b) is better in terms of sequence similarity, though is unrealistic in terms of segment coverage, which would make the matching in (c) preferable. (d) The corresponding network. Each pair of consecutive layers is a bipartite graph with capacities *c *and costs *w*' as described.

Consider arbitrary sets *X *and *Y*. A *many-to-many matching *(henceforth a *matching*) between *X *and *Y *is a set *M *of pairs {(*x*, *y*) ∈ *X *× *Y*} (see Figure 2, (a), (b), (c). The *coverage *of an element *x *∈ *X *with respect to a matching *M *is *c_M _*(*x*) = *|*{*y *: (*x*, *y*) ∈ *M*}|. Symmetrically, *c_M _*(*y*) = |{*x *: (*x*, *y*) ∈ *M*}| for *y *∈ *Y *.

A *coverage sensitive matching cost function *(henceforth a *cost function*) *w *for *X *and *Y *assigns *matching costs w_m _*(*x*, *y*) for every pair (*x*, *y*) ∈ *X *× *Y *, and *coverage costs w_c _*(*z*, *i*) for every *z *∈ *X *∪ *Y *and every integer *i ≥ *0. The *cost *of a matching *M *between *X *and *Y *with respect to *w *is given by

(1) $$w\left(M\right)=\underset{\left(x,y\right)\in M}{\sum }w_{m}\left(x,y\right)+\underset{z\in X\cup Y}{\sum }w_{c}\left(z,c_{M}\left(z\right)\right)$$

### The CSM problem

**Input: **A Matching Instance (*X*, *Y*, *w*) consisting of sets *X*, *Y*, and cost function *w*.

**Output: **Compute $CSM\left(X,Y,w\right)=\underset{M\subseteq X\times Y}{\text{min}}w\left(M\right)$.

Note that CSM is a generalization of classical problems in combinatorics. For example, consider the problem of finding a maximum (partial one-to-one) matching on a bipartite graph *G *with vertex shores *X*, *Y*, and an edge set *E*. This problem can be solved by solving CSM on the input *X*, *Y *using the following costs: set *w_c _*(*z*, 0) = *w_c _*(*z*, 1) = 0, and *w_c _*(*z*, *i*) = ∞ for all *z *∈ *X *∪ *Y*, *i >*1; set *w_m _*(*x*, *y*) = -1 for (*x*, *y*) ∈ *E *and otherwise set *w_m _*(*x*, *y*) = ∞. Similarly, CSM can also be used for solving the minimum/maximum weight variants of the bipartite matching problem. However, CSM is NP-hard in general (see Additional File 1), and therefore we do not expect to solve the general instance efficiently.

### CSM with convex coverage costs

Let (*X*, *Y*, *w*) be a matching instance. We say that *w *has *convex *coverage costs if for every element *z *∈ *X *∪ *Y *and every integer *i >*0, $w_{c}\left(z,i\right)\leq \frac{w_{c}\left(z,\;\;i-1\right)\;+\;w_{c}\left(z,\;\;i+1\right)}{2}$. We show here that *CSM *with convex coverage costs can be reduced to the poly-time solvable *min-cost integer flow *problem [11].

For *x *∈ *X*, denote *d_x _*= |{*y *: *w_m _*(*x*, *y*) *<*∞}|, and similarly *d_y _*= *|*{*x *: *w_m _*(*x*, *y*) *<*∞}| for *y *∈ *Y *. Denote $d_{X}=\underset{x\in X}{\text{max}\;}d_{x}$ and $d_{Y}=\underset{y\in Y}{\text{max}\;}d_{y}$. The reduction builds the flow network *N *= (*G*, *s*, *t*, *c*, *w*'), where *G *is the network graph, *s *and *t *are the source and sink nodes respectively, and *c *and *w*' are the edge capacity and cost functions respectively. The graph *G *= (*V*, *E*) is defined as follows (Figure 2d).

• *V *= *X *∪ *Y *∪ *C^X ^*∪ *C^Y ^*∪ {*s*, *t*}, where the sets $C^{X}=\left\{c_{1}^{X},c_{2}^{X},...,c_{d_{X}}^{X}\right\}$, $C^{Y}=\left\{c_{1}^{Y},c_{2}^{Y},...,c_{d_{Y}}^{Y}\right\}$, and {*s*, *t*} contain unique nodes different from all nodes in *X *and *Y *. Note that we use the same notations for elements in *X *and *Y *and their corresponding nodes in *V*, where ambiguity can be resolved by the context.

• *E *= *E*_1 _∪ *E*_2 _∪ *E*_3 _∪ *E*_4 _∪ *E*_5_, where

- $E_{1}=\left\{\left(s,\;c_{i}^{X}\right):c_{i}^{X}\in C^{X}\right\}$,

- $E_{2}=\left\{\left(c_{i}^{X},\;x\right)\;:\;c_{i}^{X}\in C^{X},\;x\in X,\;d_{x}\leq i\right\}$,

- $E_{3}=\left\{\left(x,\;y\right)\;:\;x\in X,\;y\in Y,\;w_{m}\left(x,\;y\right)<\infty \right\}$,

- $E_{4}=\left\{\left(y,\;c_{i}^{Y}\right):y\in Y,\;c_{i}^{Y}\in C^{Y},\;d_{y}\leq i\right\}$,

and

- $E_{5}=\left\{\left(c_{i}^{Y},\;t\right):c_{i}^{Y}\in C^{Y}\right\}$.

The capacity function *c *assigns infinity capacities to all edges in *E*_1 _and *E*_5 _and unit capacities to all edges in *E*_2_, *E*_3 _and *E*_4_. The cost function *w*' assigns zero costs to edges in *E*_1 _and *E*_5_, costs *w_c _*(*x*, *i*) - *w_c _*(*x*, *i *- 1) to edges $\left(c_{i}^{X},x\right)\in E_{2}$, costs *w_c _*(*y*, *i*) - *w_c _*(*y*, *i *- 1) to edges $\left(y,\;\;c_{i}^{Y}\right)\in E_{4}$, and costs *w_m _*(*x*, *y*) to edges (*x*, *y*) ∈ *E*_3_. For *E*' ⊆ *E*, denote $w'\left(E^{\prime }\right)=\underset{e\in E^{\prime }}{\sum }w'\left(e\right)$. An *integer flow *in *N *is a function *f *: *E *→ {0, 1, 2, . . .}, satisfying that *f*(*e*) ≤ *c*(*e*) for every *e *∈ *E *(*capacity constraints*), and $\underset{u:\left(u,v\right)\in E}{\sum }f\left(u,v\right)=\underset{u:\left(u,v\right)\in E}{\sum }f\left(v,u\right)$ for every *v *∈ *V *\ {*s*, *t*} (*flow conservation constraints*). The cost of a flow *f *in *N *is defined by $w'\left(f\right)\;=\underset{e\in E}{\sum }f\left(e\right)w'\left(e\right)$.

In what follows, let (*X*, *Y*, *w*) be a matching instance where *w *has convex coverage costs, and let *N *be its corresponding network. Due to the convexity requirement, for every *x *∈ *X *and every integer *i >*0, $\begin{array}{c} w'\left(c_{i+1}^{X},\;x\right)-w'\left(c_{i}^{X},\;x\right)=\left(w_{c}\left(x,\;i+1\right)-w_{c}\left(x,\;i\right)\right)-\left(w_{c}\left(x,\;i\right)-w_{c}\left(x,\;i-1\right)\right)\\ =w_{c}\left(x,\;i+1\right)+w_{c}\left(x,\;i-1\right)-2w_{c}\left(x,\;i\right)\geq 0. \end{array}$ Similarly, for every *y *∈ *Y *and every integer *i >*0, $w'\left(y,c_{i+1}^{Y}\right)-w'\left(y,c_{i}^{Y}\right)\geq 0$, and we get the following observation:

**Observation 1**. *Series of the form *$w'\left(c_{1}^{X},\;x\right),w'\left(c_{2}^{X},\;x\right),\;...$ and $w'\left(y,\;c_{1}^{Y}\right),w'\left(y,\;c_{2}^{Y}\right),\;...$*are non-decreasing. Consequentially, for every *$E^{\prime }\subseteq \left\{\left(c_{i}^{X},\;x\right)\;:\;x\in X,\;1\leq i\leq d_{x}\right\}$*and *$E^{\prime \prime }=\left\{\left(c_{i}^{X},\;x\right)\;:\;x\in X,\;1\leq i\leq |E^{\prime }|\right\},$$w'\left(E^{\prime \prime }\right)\leq w'\left(E^{\prime }\right)$, *and similarly for *$E^{\prime }\subseteq \left\{\left(y,\;c_{i}^{Y}\right)\;:\;y\in Y,\;1\leq i\leq d_{y}\right\}$*and *$E^{\prime \prime }=\left\{\left(y,\;c_{i}^{Y}\right)\;:\;y\in Y,\;1\leq i\leq |E^{\prime }|\right\}$.

Given a flow *f *in *N*, define the matching *M_f _*= {(*x*, *y*) : (*x*, *y*) ∈ *E*_3_, *f*(*x*, *y*) = 1}. Denote $E_{x}^{f}=\left\{\left(c_{i}^{X},x\right):f\left(c_{i}^{X},x\right)=1\right\}$ and $E_{y}^{f}=\left\{\left(y,\;c_{i}^{Y}\right):f\left(y,\;\;c_{i}^{Y}\right)=1\right\}$. Since for edges *e *∈ *E*_1 _∪ *E*_5 _we have that *w*'(*e*) = 0, and since for edges *e *∈ *E*_2 _∪ *E*_3 _∪ *E*_4 _we have that *f*(*e*) ∈ {0, 1} (due to capacity constraints), we can write

(2) $$\begin{array}{llll} w'\left(f\right) & =\underset{e\in E}{\sum }f\left(e\right)w'\left(e\right)=\underset{\begin{array}{c} e\in E_{2}\cup E_{3}\cup E_{4}\\ \;\;\;\;\;\;\;\;f\left(\text{e}\right)=1 \end{array}}{\sum }w'\left(e\right)\; & & \;\\ & =w'\left(M_{f}\right)+\underset{x\;\in \;X}{\sum }w'\left(E_{x}^{f}\right)+\underset{y\;\in \;Y}{\sum }w'\left(E_{y}^{f}\right).\; & & \;\\ & \; & \end{array}$$

Given a non-infinity cost matching *M *between *X *and *Y*, define the flow *f_M _*in *N *as follows:

• For every (*x*, *y*) ∈ *E*_3_, *f *(*x*, *y*) = 1 if (*x*, *y*) ∈ *M*, and otherwise *f*(*x*, *y*) = 0;

• For every $\left(c_{i}^{X},\;x\right)\in E_{2},\;f\left(c_{i}^{X},\;x\right)=1$ if *c_M _*(*x*) ≤ *i*, and otherwise $f\left(c_{i}^{X},\;x\right)=0$;

• For every $\left(y,\;c_{i}^{Y}\right)\in E_{4}$, $f\left(y,\;c_{i}^{Y}\right)=1$ if *c_M _*(*y*) ≤ *i*, and otherwise $f\left(y,\;c_{i}^{Y}\right)=0$;

• For every $\left(s,\;c_{i}^{X}\right)\in E_{1},\;f\left(s,\;c_{i}^{X}\right)=|\left\{x:f\left(c_{i}^{X},\;x\right)=1\right\}|$;

• For every $\left(c_{i}^{Y},\;t\right)\in E_{5},\;f\left(c_{i}^{Y},\;t\right)=\;|\left\{y:f\left(y,\;c_{i}^{Y}\right)=1\right\}|$.

It is simple to assert that *f_M _*is a valid flow in *N *(satisfying all capacity and flow conservation constraints), and that $M_{f_{M}}=M$.

**Claim 1**. *For every flow f in N*, $w'\left(f_{M_{f}}\right)\leq w'\left(f\right).$

*Proof*. From flow conservation constraints $|E_{x}^{f}|=|E_{x}^{f_{M_{f}}}|=c_{M_{f}}\left(x\right)$ for every *x *∈ *X*, where in particular by definition we have that $E_{x}^{f_{M_{f}}}=\left\{\left(c_{i}^{X},\;x\right):1\leq i\leq c_{M_{f}}\left(x\right)\right\}$ Therefore, it follows from Observation 1 that $w'\left(E_{x}^{f_{M_{f}}}\right)\leq w'\left(E_{x}^{f}\right)$ for every *x *∈ *X*, and similarly it may be shown that $w'\left(E_{x}^{f_{M_{f}}}\right)\leq w'\left(E_{x}^{f}\right)$ for every *y *∈ *Y*. Hence,

$$\begin{array}{llll} w'\left(f_{M_{f}}\right) & \;\overset{\text{Eq}.2}{=}\;w'\left(M_{f_{M_{f}}}\right)+\underset{x\in X}{\sum }w'\left(E_{x}^{f_{M_{f}}}\right)\; & & \;\\ & \;\;\;+\underset{y\in Y}{\sum }w'\left(E_{y}^{f_{M_{f}}}\right)\; & & \;\\ & \;\leq \;w'\left(M_{f}\right)+\underset{x\in X}{\sum }w'\left(E_{x}^{f}\right)\; & & \;\\ & \;\;\;+\underset{y\in Y}{\sum }w'\left(E_{y}^{f}\right)\; & & \;\\ & \;\overset{\text{Eq}.2}{=}\;w'\left(f\right).\; & & \; \end{array}$$

□

Denote $\Delta =\Delta \left(X,\;Y,\;w\right)=\underset{z\in X\cup Y}{\sum }w_{c}\left(z,\;0\right)$, and note that Δ depends only on the instance (*X*, *Y*, *w*) and not on any specific matching.

**Claim 2**. *For every matching M between × and Y, w*'(*f_M_*) = *w*(*M*) - Δ.

*Proof*. For *x *∈ *X*, we have that $\begin{array}{c} w'\left(E_{x}^{f_{M}}\right)=w'\left(c_{1}^{X},\;x\right)+w'\left(c_{2}^{X},\;x\right)+...+w'\left(c_{c_{M}\left(x\right)}^{X},\;x\right)\\ =\left(w_{c}\left(x,\;1\right)-w_{c}\left(x,\;0\right)\right)+\left(w_{c}\left(x,\;2\right)-w_{c}\left(x,\;1\right)\right)+...+\left(w_{c}\left(x,\;c_{M}\left(x\right)\right)\;-\;w_{c}\left(x,\;c_{M}\left(x\right)-1\right)\right)\\ =w_{c}\left(x,\;c_{M}\left(x\right)\right)-w_{c}\left(x,\;0\right), \end{array}$ and similarly $w'\left(E_{y}^{f_{M}}\right)=w_{c}\left(y,\;c_{M}\left(y\right)\right)-w_{c}\left(y,\;0\right)$ for *y *∈ *Y*. Therefore,

$$\begin{array}{llll} w'\left(f_{M}\right) & \;\overset{\text{Eq}.2}{=}\;w'\left(M\right)+\underset{x\in X}{\sum }w'\left(E_{x}^{f_{M}}\right)\; & & \;\\ & \;\;\;+\underset{y\in Y}{\sum }w'\left(E_{y}^{f_{M}}\right)\; & & \;\\ & \;\;\;=w'\text{(}M\text{)}\; & & \;\\ & \;\;\;+\underset{x\in X}{\sum }\left(w_{c}\left(x,\;\;c_{M}\left(x\right)\right)-w_{c}\left(x,\;0\right)\right)\; & & \;\\ & \;\;\;+\underset{y\in Y}{\sum }\left(w_{c}\left(y,\;c_{M}\left(y\right)\right)-w_{c}\left(y,\;0\right)\right)\; & & \;\\ & \;\;\;=\underset{\left(x,y\right)\in M}{\sum }w_{m}\left(x,\;y\right)\; & & \;\\ & \;\;\;+\;\underset{z\in X\cup Y}{\sum }w_{c}\left(z,\;c_{M}\left(z\right)\right)\; & & \;\\ & \;\;\;-\underset{z\in X\cup Y}{\sum }w_{c}\left(z,\;0\right)\; & & \;\\ & \;\overset{\text{Eq}.1}{=}\;w\left(M\right)-\Delta .\; & & \; \end{array}$$

□

**Claim 3**. Let f* be a minimum cost flow in N. Then, M_f* _is a minimum cost matching between X and Y, and CSM(*X*, *Y*, *w*) = *w*'(f*) + Δ.

*Proof*. Since f* is a minimum cost flow in *N*, $\;w'\left(f^{*}\right)\leq w'\left(f_{M_{f^{*}}}\right)\overset{\text{Clm}.1}{\leq }w'\left(f^{*}\right)$, thus $w'\left(f^{*}\right)=w'\left(f_{M_{f^{*}}}\right)$. Let *M *be a matching between *X *and *Y*. Again, from the optimality of f*, *w*'(f*) ≤ *w*'(*f_M_*) and so $w\left(M_{f^{*}}\right)-\Delta \;\overset{Clm.2}{=}w'\left(f_{M_{f*}}\right)=w'\left(f^{*}\right)\leq w'\left(f_{M}\right)\overset{Clm.2}{=}w\left(M\right)-\Delta$, and in particular $w\left(M_{f^{*}}\right)\leq w\left(M\right)$. Thus, $M_{f^{*}}$ is a minimum cost matching for (*X*, *Y*, *w*), and so $CSM\left(X,\;Y,\;w\right)=w\left(M_{f^{*}}\right)\overset{Clm.2}{=}w'\left(f^{*}\right)+\Delta$.

□

## Constructing CSM instance from read mapping data

Consider a set of reads and a prediction of the genomic sequence (henceforth, the "prediction") from which the reads were extracted. It is assumed that the sequencing procedure produces reads with some sequencing error probability, and that read extraction positions along the genome adhere to some expected distribution. The probability for extracting a read starting at a given position may depend on the sequential context at this position and its location along the genome. Given such probabilities, it is possible to compute for a given segment of the prediction an expected amount of extracted reads starting within this segment. Such an amount of expected reads will be referred to here as the *expected coverage *of the segment. Hence, we can argue that the reads *well support *the prediction in case it is possible to assign to each read a position within the prediction, from which it was presumably extracted, in a manner that (a) each read sequence approximately matches the substring of the prediction starting at the assigned position, and (b) for every segment of the prediction, the amount of reads assigned to positions within this segment does not deviate significantly from the expected coverage of the segment. On the other hand, when no such position assignment can be found, it is suggestive that the prediction exhibits some variation with respect to the true genome.

Given a predicted region, a *mapping *between the reads and the prediction is a function that assigns to each read a set of positions in the region from which it is possible to extract the read (with some allowed amount of sequencing errors). Software tools for producing such mappings exist (e.g. Bowtie [8]) and are widely used. Ideally, if the prediction is in fact the correct genomic sequence from which the reads were extracted, and this region is non-repetitive, it is expected that a mapping would assign to each read a unique position that is the true position from which it was extracted. Nevertheless, when the sequence contains repeats, and sequencing errors are not negligible, it is expected that some of the reads will be mapped to multiple positions (due to the repeats), while others may not be mapped to any position (due to sequencing errors). Given a mapping between the reads and the region, we define a *read-to-genome matching *as a function that selects for each read at most one corresponding position among its set of positions given by the mapping, from which it was presumably extracted. A read-to-genome matching better supports the prediction the more reads it matches to the genome, the higher the similarity is between reads and their matching positions, and the smaller the deviation is between the expected coverage and the coverage implied by the matching positions.

The quality of a read-to-genome matching can be naturally evaluated using the CSM formalism described in the previous section. A matching instance (*X*, *Y*, *w*) can be generated, choosing *X *to be the set of reads, and *Y *to be a partition of the prediction into segments (where each element in *Y *corresponds to a segment in the partition). For each read *x *∈ *X *and each segment *y *∈ *Y*, *w_m _*(*x*, *y*) is set to the best sequence similarity score between *x *and a substring of the prediction starting at *y *(such similarity scores may be generated by tools such as Bowtie [8]), or set to ∞ if no substring starting at *y *is similar to *x*. The coverage cost function for a read *x *∈ *X *sets *w_c _*(*x*, 0) to some penalty added to the score in case *x *is unmatched, sets *w_c _*(*x*, 1) to 0 (no penalty is added when *x *participates in the matching), and *w_c _*(*x*, *i*) for *i >*1 to ∞ (a matching in which a read is assigned to more than one position is illegal, and has an infinite cost). For a segment *y *∈ *Y*, it is possible to compute the expected coverage *c_y _*of *y*, and generate a convex score function *f*(*i*) whose minimum point is at *i *= *c_y_*, and set *w_c _*(*y*, *i*) = *f *(*i*) for every nonnegative integer *i*. The cost of an optimal matching for this instance can then serve as a quality measure for the prediction.

### Implementation

We implemented the CSM algorithm as a java based tool named SAGE, a **S**coring function for **A**ssembled **GE**nomes. The inputs to SAGE are a set of reads, *R*, mapped to a genomic template, *G*, in the BAM format [16] along with a parameter file containing alignment costs, unmatched read penalty, genome segmentation, expected segment coverage values, and a choice of coverage cost functions (currently linear and polynomial cost functions).

## Results

We tested SAGE on the hypervariable KIR region. The KIR region, while variable, is tightly organized and contains between 8 and 14 genes, and 2 pseudo-genes (Figure 1a) [17]. The genes are organized into two adjacent regions, each bordered by two anchoring genes/pseudo-genes: KIR3DL3 and 3DP1 for the centromeric region; 2DL4 and 3DL2 for the telomeric region. Variability within KIR is expressed in the form of changing gene numbers, gene-copy numbers, and gene polymorphisms. There are two broad types of KIR haplotypes- Type A and Type B- that are distinguished by their gene content. Type A haplotypes are characterized by the absence of the following genes: {KIR-2DL5, -2DS1, -2DS2, -2DS3, -2DS5, -3DS1}, while Type B haplotypes contain one or more of these genes [18]. Type B haplotypes can be split further into different sub-types, characterized by the gene content on the centromeric-side and telomeric-side. The various (sub-)types of KIR haplotypes are denoted by {A, AB, BA1, BA2, BA2X, Bdel, B}. However, the typing is incompletely developed, and is likely to change as more data is acquired.

To test the effectiveness of SAGE on a variety of haplotype types, we simulated reads from 27 known KIR haplotypes using GemSIM [19] with an error model learned from paired-end (100 × 2)bp reads generated by Illumina GA IIx with TrueSeq SBS Kit v5-GA [19]. The 27 haplotype templates were taken from the IPD-KIR database [20]. The sequences of these templates were obtained experimentally by first separating the two haplotypes of an individual using fosmid-pools, determining the gene content and architecture of each haplotype using STS assays, and then finally sequencing the individual fosmids [21].

Before we ran SAGE, we mapped each read set, *R*, back to each template, *G*, using Bowtie. We ran Bowtie under the '-a' option with all other parameters set to the default, in order to obtain a set of all possible mapping locations and their corresponding alignment costs for each read, which was used as input into SAGE. The mapping position of a paired-end read was set to be the genomic index to which the first character of the first sub-read was aligned. The alignment cost for a complete (100 × 2)bp paired-end read varied between 0 and 180, with 0 corresponding to identity. When two paired-ends mapped in a concordant manner, the total alignment cost for the read was calculated by adding the alignment cost of both paired-ends. When a paired-end did not have a concordant mate, suggestive of incorrect architecture, the alignment cost was further penalized by adding a cost of 90, which is the maximum penalty for one paired-end. The unmatched read penalty was constant for all reads and set to 100. On the other side, the genome *G *was partitioned to segments of fixed length of 1000bp (except for the last segment which may be shorter), with expected coverage per segment given by $\lambda =1000\frac{\left|R\right|}{\left|G\right|}$ (with the appropriate adjustment for the last segment), where |*R*| and |*G*| denote the number of reads and the length of the genome, respectively. To allow for natural variation in coverage, the quadratic function, *f*(*i*) = (λ - *i*)^2^, was chosen as the segment coverage cost function.

To the best of our knowledge, SAGE is the first tool that scores templates given a set of reads. As there is no competing tool, we compared SAGE results against a naive approach that ignores coverage and sums up the best alignment score for each read to obtain a total score for each read set and template. The scores obtained by this approach will be referred to as the *Bowtie scores *below.

### Haploid templates

**Figure 3:**
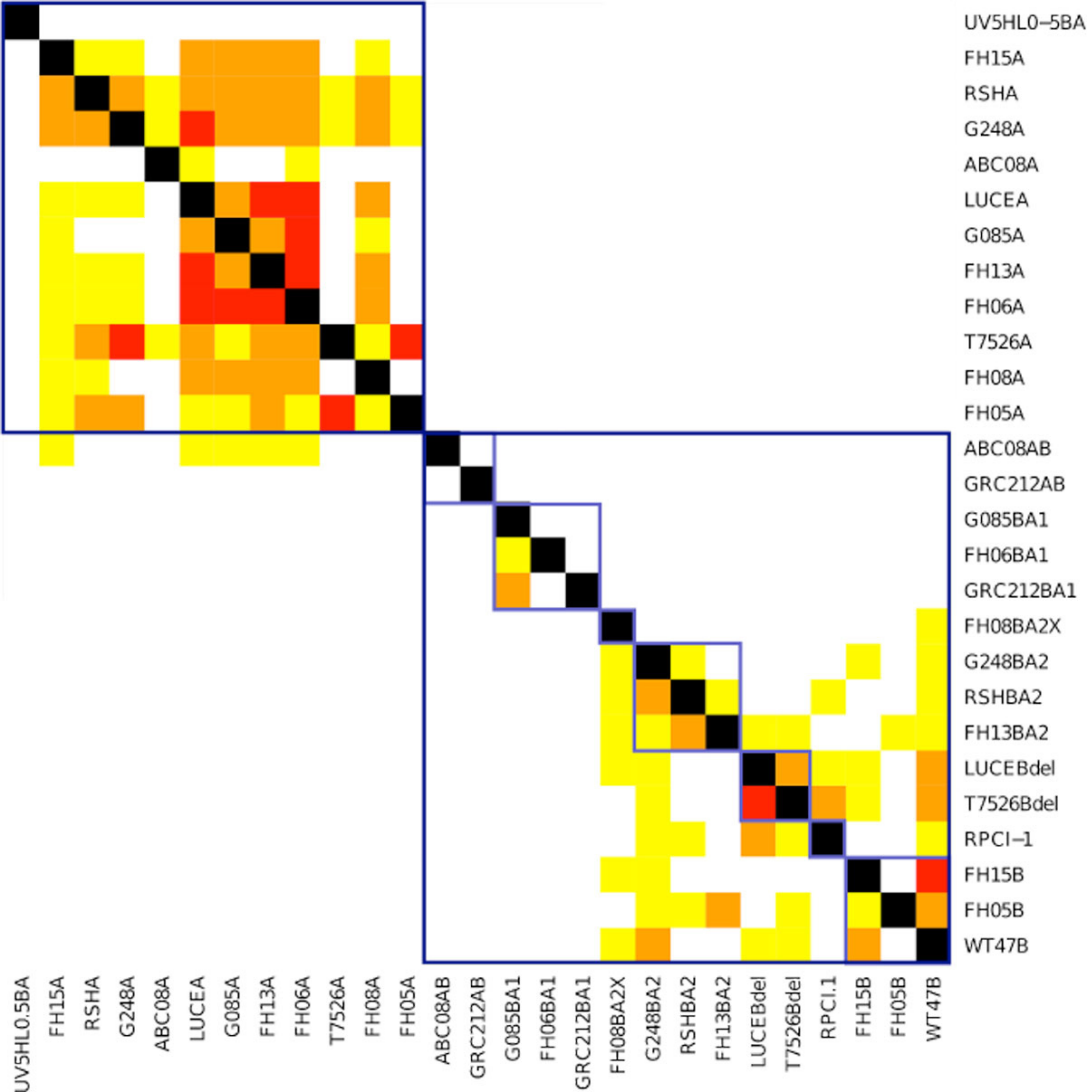
**Scoring simulated reads against haploid templates using SAGE**. Each row contains the color-coded percentage from the top-score of a read-set mapped against 27 genomic templates. Black: top-score; Red: within 5% of top-score; Orange: ≤ 10%; Yellow: ≤ 20%; White: *>*20% below top-score. Sequences are ordered along the rows and columns so that sequences with the same (sub-)type are adjacent to each other. Templates of the same type are indicated by the blue boxes, and those of the same sub-type by light blue boxes.

As a first pass, we tested SAGE's ability to score haploid templates. We scored each of the 27 read sets against each of the 27 templates using SAGE. A visualization of the scores are shown in Figure 3, where the templates are organized by sequence similarity so that templates of the same type/sub-type are clustered together. Note that the matrix is not symmetric. Each row corresponds to the scores of a single read data set against a collection of haploid templates. As can be seen, SAGE always gets the top-score for the correct template. Moreover, the other templates from the same sub-type get progressively weaker scores. Major haplotypes fall within distinct blocks, but the scores also suggest a hierarchy within the subtypes that can be studied further.

### Dipolid templates

**Figure 4:**
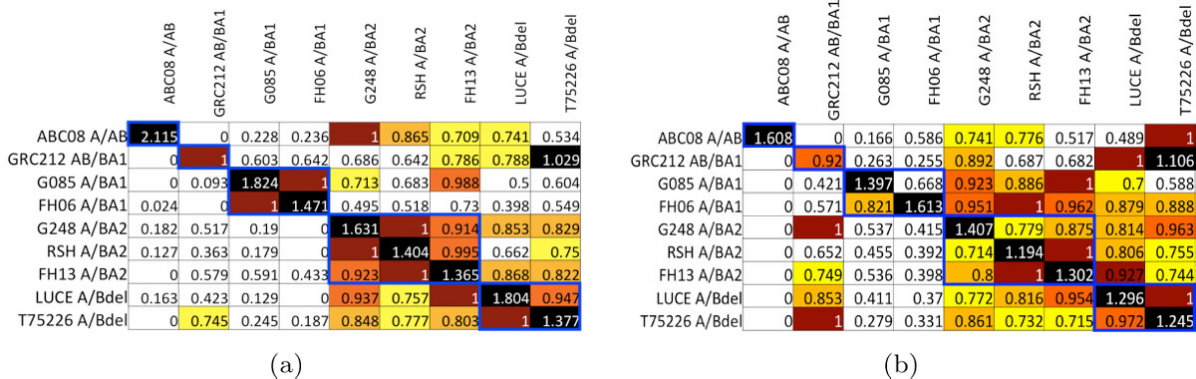
**Scoring simulated reads against diploid templates**. Each row of a matrix represent scores from the same read sets mapped to different prediction templates. The scores are normalized so that the *second best *score in each row is equal to 1 and the worst score is equal to 0. We normalize with respect to the second best score since it would be used to predict the haplotype in the absence of the best scoring (and presumably correct) template. Furthermore, the entries are color-coded accordingly- Black: top-score; Red: second top-score; Orange: within 10% of second top-score; Light Orange: ≤ 20%; Yellow: ≤ 30%; White: *>*30% below top-score. Both matrices are ordered according to template sub-types. Templates of the same type are indicated by the blue boxes. (a) SAGE scores (b) Bowtie scores.

To test scoring on more realistic templates, we simulated reads from 9 diploid individuals whose pair of haploid templates were obtained experimetally in Pyo *et al*. [21] and are in the IPD-KIR database [20]. The 9 diploid templates from this study fell into one of 6 combination of sub-types. We scored each of the 9 simulated read sets against each of the 9 diploid templates using SAGE. In all but one case, SAGE (Figure 4a) and Bowtie (Figure 4b) predicted the correct diploid template of the donor. Furthermore, SAGE is better at predicting the sub-type of the donor template than Bowtie. When the donor template is not in database, as is usually the case in practice, SAGE will give a better score to templates that are more similar to the donor while Bowtie may not. For example, row 3 of Figure 4(a, b) show the scores when the donor template is of type A and BA1. Both SAGE and Bowtie correctly gave the best score to the diploid template G085-A/BA1. However, the template with the next best SAGE score was also of sub-type A/BA1, while the template with the next best Bowtie score was of subtype A/BA2.

**Figure 5:**
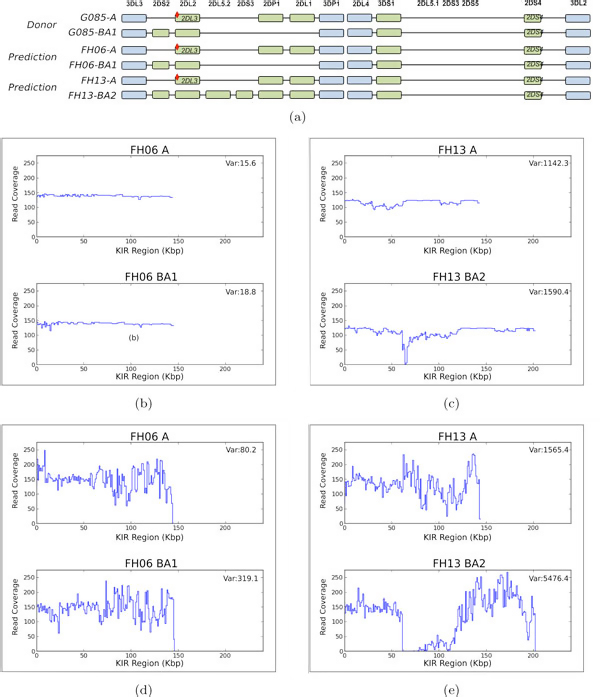
**Coverage plots for reads sampled from G085-A/BA1 templates**. (a) genomic architecture of of G085-A/BA1, FH06-A/BA1, and FH13-A/BA2. SAGE coverage plots when reads are extracted from G085A/BA1 and mapped to (b) FH06-A/BA1 and (c) FH13 A/BA2. Bowtie coverage plots when reads are extracted from G085A/BA1 and mapped to (d) FH06-A/BA1 and (e) FH13-A/BA2.

In general, coverage plays an important role in determining the correct haplotype. Figure 5(b-e) show the coverage plots when reads from donor template G085-A/BA1 are mapped to a template of the same sub-type (F06-A/BA1) and a template of a different sub-type (FH13-A/BA2) using SAGE and Bowtie. When mapped to templates of the same sub-type (Figure 5(b, d)), the coverage plots for both SAGE and Bowtie show less variance when compared to the coverage plots of the other templates (Figure 5(c, e)). Bowtie does not take into account variance of coverage and scores the template of a different sub-type (FH13-A/BA2) higher than the template of the same sub-type (F06-A/BA1). On the contrary, SAGE penalizes for the variance in coverage, and correctly predicts the sub-type of the donor. Furthermore, if several possible mappings of a read are given, SAGE can be used to determine the best mapping. In Figure 5(b, c), we see less variability in the coverage plots from SAGE's matching compared against those of Bowtie's matching (Figure 5(d, e)). Therefore, even if Bowtie is able to determine the correct donor template, it may not output the correct mapping.

### Running time

For a data-set with *n *reads and a total of *m *read mapping locations, SAGE scales as *O*(*nm *+ *n*^2 ^log *n*). Thus, on our data-sets with haploid genomes of average length 166Kbp (166 1000bp-segments), and ~ 24,900 reads, SAGE ran in 21 seconds. The running time increased to 210 seconds for the average diploid genome (~ 332 1000bp-segments, ~ 49,800 reads). Running times were recorded using a 4 core Intel 2.66GHz processor with 9Gb of RAM.

## Discussion and conclusions

To the best of our knowledge, SAGE is the first tool that scores predicted donor templates given a set of sequenced reads. Our results on the KIR region show that SAGE can be used to predict the sub-type of the donor KIR template, and can be directly used for haplotyping this region. Furthermore, SAGE scores the correct template higher than even templates of the same sub-type.

While we focused our attention on the KIR region, SAGE is general enough to be applied to any complex region. It is also possible to implement many different scoring functions, which would allow the user to obtain optimal matchings according to his own custom scores. For example, read unmatching penalties may be constant for all reads, or may be read-specific. A motivation for read specific costs is in the case where the sequencing phase produces some sequencing qualities for reads, and it is possible to "pay" less when not matching reads of lower sequencing quality. Similarly, it is possible to choose a segmentation of the prediction in which all segments are of the same length, and uniform coverage is assumed, or one with variable segment lengths and possibly different coverage cost functions for each segment. A motivation to such complex segmentation is e.g. in the case where one tries to identify a specific structural variation, such as a deletion of a segment of specific length around a specific region of the prediction. Setting lengths of segments in the examined region to the expected deletion length can increase the likelihood that an optimal matching would not add artifact matchings of reads to a long segment spanning the deleted segment, in order to compensate for low coverage of the deleted segment. Lastly, by using different coverage cost functions, it is possible to decide the rate in which penalty increases due to deviations of expected coverage, which may grow linearly, polynomially, exponentially, or based on other probabilistic models, as long as the function satisfies the convexity requirement.

Future work would involve extending the use of SAGE on real data. Some challenges in dealing with real data include obtaining the set of reads extracted from the region of interest (especially when sequencing data is likely taken from the whole genome) and providing the expected coverage. If we know the parameters of the sequencing run, we could use the target read coverage as the expected coverage; however, if that is unknown, we may be able to estimate the expected coverage from the number of reads we need to map to the region. For example, if we assume a uniform distribution of coverage, then the expected coverage per segment is simply the total length of the segment multiplied by $\frac{\left|R\right|}{\left|G\right|}$.

Although haplotype analysis of the KIR region is medically relevant, the genomic complexity (i.e. repetitive nature and variable gene architecture) of this region makes it difficult to do a complete analysis. Indeed, the possible sub-types of this region have not been completely characterized. Thus, reconstruction of this region and other complex regions of the genome remain a worthwhile problem. SAGE takes the first step in reconstructing complex regions of the genome by providing a scoring function for predicted templates based on their similarity to the true donor. Therefore, it might be possible to obtain a complete reconstruction of the donor genome by iteratively refining predicted donor templates until SAGE scores are optimized. Furthermore, SAGE can also be applied for scoring *de-novo *assemblies and for comparing the accuracies of different assemblers.

## List of abbreviations used

CSM: Coverage Sensitive many-to-many min-cost Matching; SAGE: Scoring function for Assembled Genomes; KIR: Killer cell Immunoglobulin-like Receptor; HLA: Human Leucocyte Antigen

## Competing interests

The authors declare that they have no competing interests.

## Authors' contributions

SZ and CL developed and implemented the method. CL and SK designed the experiments. CL performed the experiments. CL, SZ, and VB wrote the manuscript. All authors read and approved the final manuscript.

## Supplementary Material

Additional file 1 **NP-hardness of CSM**. Click here for file

## Declarations

Publication of this article was supported by NIH (RO1-HG004962, U54 HL108460) and NSF (CCF-1115206).

This article has been published as part of *BMC Bioinformatics *Volume 14 Supplement 5, 2013: Proceedings of the Third Annual RECOMB Satellite Workshop on Massively Parallel Sequencing (RECOMB-seq 2013). The full contents of the supplement are available online at http://www.biomedcentral.com/bmcbioinformatics/supplements/14/S5.
