# Supplementary material for: Evaluating genome architecture of a complex region via generalized bipartite matching
Source: BMC Bioinformatics. 2013 Apr 10;14(Suppl 5):S13. doi: 10.1186/1471-2105-14-S5-S13 (PMC3622632; doi:10.1186/1471-2105-14-S5-S13)
Supplement: Additional file 1 — NP-hardness of CSM. [file 1471-2105-14-S5-S13-S1.PDF]

NP-Hardness of CSM

In this section we show that for general (non-convex) scoring functions, the CSM problem is NP-Hard. This is proven by reducing the NP-Hard problem SAT to CSM.

Let $B = \{b_1, b_2, \ldots, b_n\}$ be a set of boolean variables. An *assignment* for B is a function $A : B \to \{true, false\}$. A *CNF clause* ϕ over B is a boolean clause of the form $\phi = (x_1 \vee x_2 \vee \ldots \vee x_k)$, where each literal x_i is either some variable $b \in B$, or a negation $\neg b$ of some variable $b \in B$. For an assignment A for B and a variable $b \in B$, define $A(\neg b) = \neg A(b)$. The clause ϕ is *satisfied* by A if $A(x) = true$ for at least one literal x appearing in ϕ. A *CNF formula* ψ over B is of the form $\psi = \phi_1 \wedge \phi_2 \wedge \ldots \wedge \phi_m$, where each ϕ_j appearing in ψ is a CNF clause. The CNF formula ψ is *satisfied* by an assignment A if all clauses in ψ are satisfied by A. Say that ψ is *satisfiable* if there exists some satisfying assignment for ψ. The CNF-SAT problem is, given a CNF formula ψ over a set of variables B, to decide whether ψ is satisfiable. CNF-SAT is a well known NP-Complete problem [**?**, **?**]. Next, we show that CNF-SAT can be reduced to CSM in a polynomial time, proving NP-Harness of CSM.

Given a CNF formula $\psi = \phi_1 \wedge \phi_2 \wedge \ldots \wedge \phi_m$ over the set of variables $B = \{b_1, b_2, \ldots, b_n\}$, the reduction constructs the matching instance (X, Y, w) as follows:

- The set X contains an element for each literal of B, i.e. $X = \{b_1, \neg b_1, b_2, \neg b_2, \ldots, b_n, \neg b_n\}$.
- The set Y is the union of two subsets $Y^b = \{y^b_1, y^b_2, \ldots y^b_n\}$ and $Y^\phi = \{y^\phi_1, y^\phi_2, \ldots y^\phi_m\}$. Each element $y^b_i \in Y^b$ corresponds to a variable $b_i \in B$, and each element $y^\phi_j \in Y^\phi$ corresponds to a clause ϕ_j of ψ.
- The cost function w defines the following matching and coverage costs:
  - For each $1 \le i \le n$, set $w_m\left(b_i, y^b_i\right) = w_m\left(\neg b_i, y^b_i\right) = 0$, and $w_m\left(x, y^b_i\right) = 1$ for $x \notin \{b_i, \neg b_i\}$. In addition, set $w_m\left(x, y^\phi_j\right) = 0$ if the literal x appears in ϕ_j, and otherwise set $w_m\left(x, y^\phi_j\right) = 1$.
  - For each $x \in X$, let d_x be the number of clauses in ψ containing the literal x. Set $w_c(x, 0) = w_c(x, d_x + 1) = 0$, and $w_c(x, a) = 1$ for $a \notin \{0, d_x + 1\}$. In addition, $w_c\left(y^b_i, 1\right) = 0$ and $w_c\left(y^b_i, a\right) = 1$ for $a \neq 1$, and $w_c\left(y^\phi_j, 0\right) = 1$ and $w_c\left(y^\phi_j, a\right) = 0$ for $a \ge 1$.

Claim 1. *ψ is satisfiable if an only if CSM$(X, Y, w) = 0$.*

Proof. For the first direction of the proof, assume that ψ is satisfiable, and let A be a satisfying assignment for ψ. We show that in this case CSM$(X, Y, w) = 0$.

Construct the matching M between X and Y by adding for each $x \in X$, such that $x = b_i$ or $x = \neg b_i$ and $A(x) = true$, the pair (x, y^b_i), as well as every pair (x, y^ϕ_j) such that x appears in ϕ_j. By the reduction design, for each $(x, y) \in M$, $w_m(x, y) = 0$. In addition, it is straightforward to observe that for every $x \in X$ we have that either $c_M(x) = 0$ (when $A(x) = false$) or $c_M(x) = d_x + 1$ (when $A(x) = true$), and therefore from the reduction design $w_c(x, c_M(x)) = 0$. Moreover, for each $y^b_i \in Y^b$ we have that $c_M\left(y^b_i\right) = 1$ (since M contains exactly one pair among (b_i, y^b_i) and $(\neg b_i, y^b_i)$, and no other pair in which y^b_i participates), and for each $y^\phi_j \in Y^\phi$ we have that $c_M\left(y^\phi_j\right) \ge 1$ (since ϕ_j is satisfied by A and thus M contains at least one pair of the form (x, y^ϕ_j)). Therefore, from the reduction design, $w_c(y, c_M(y)) = 0$ for every $y \in Y$, and we get that $w(M) = \sum_{(x,y) \in M} w_m(x, y) + \sum_{z \in X \cup Y} w_c(z, c_M(z)) = 0$, and in particular CSM$(X, Y, w) \le 0$. Since all costs defined by w are either 0 or 1, it is clear that CSM$(X, Y, w) \ge 0$, and thus CSM$(X, Y, w) = 0$.

For the other direction of the proof, assume that CSM$(X, Y, w) = 0$, and let M be an optimal matching between X and Y for which $w(M) = 0$. We show that in this case ψ is satisfiable.

Construct the assignment A for B, where $A(b_i) = true$ if and only if $(b_i, y_i^b) \in M$. Since $w(M) = \sum_{(x,y)\in M} w_m(x,y) + \sum_{z\in X\cup Y} w_c(z, c_M(z)) = 0$, we have that $w_m(x,y) = 0$ for every $(x,y) \in M$, and $w_c(z, c_M(z)) = 0$ for every $z \in X \cup Y$ (since all matching and coverage costs defined by w are non-negative). In particular, M contains only pairs of the form (b_i, y_i^b), $(\neg b_i, y_i^b)$, and (x, y_j^ϕ) such that x appears in ϕ_j (all other pairs have a matching cost of 1). In addition, for every $y_i^b \in Y^b$, in order to get $w_c\left(y_i^b, c_M\left(y_i^b\right)\right) = 0$ it must hold that $c_M\left(y_i^b\right) = 1$ (from the definition of w_c) and thus M contains exactly one of the pairs (b_i, y_i^b) or $(\neg b_i, y_i^b)$. This implies that for $x = b_i$ or $x = \neg b_i$, $(x, y_i^b) \in M$ if and only if $A(x) = true$. Next, for every $y_j^\phi \in Y^\phi$, in order to get $w_c\left(y_j^\phi, c_M\left(y_j^\phi\right)\right) = 0$ it must hold that $c_M\left(y_j^\phi\right) \geq 1$ (from the definition of w_c), therefore M contains at least one pair of the form (x, y_j^ϕ) such that x appears in ϕ_j^2. For such a literal x, $c_M(x) > 0$, and to obtain $w_c(x, c_M(x)) = 0$ it must hold that $c_M(x) = d_x + 1$. Hence, M must contain (x, y_i^b) and all d_x pairs of the form $(x, y_{j'}^\phi)$ such that x appears in $\phi_{j'}$, and in particular $A(x) = true$, and ϕ_j is satisfied by A. As all clauses in ψ are satisfied by A, ψ is satisfiable.

□

It is immediate to observe that the reduction described above is polynomial, and since CNF-SAT is NP-Hard it follows that CSM is NP-Hard. It is also simple to formulate CSM as a decision problem (asking whether (X, Y, w) has a matching with cost of at most k for some argument k) and to design a non-deterministic polynomial time algorithm for it (which choses a matching M at random and checks whether $w(M) \leq k$), proving that CSM is NP-Complete.
